# Two new species of *Trivalvaria* (Annonaceae) from northern Myanmar

**DOI:** 10.3897/phytokeys.94.21553

**Published:** 2018-01-29

**Authors:** Bin Yang, Shi-Shun Zhou, Hong-Bo Ding, Ren Li, Kyaw Win Maung, Yun-Hong Tan

**Affiliations:** 1 Southeast Asia Biodiversity Research Institute, Chinese Academy of Sciences, Yezin, Nay Pyi Taw 05282, Myanmar; 2 Centre for Integrative Conservation, Xishuangbanna Tropical Botanical Garden, Chinese Academy of Sciences, Menglun, Mengla, Yunnan 666303, P. R. China; 3 Forest Research Institute, Forest Department, Ministry of Environmental Conservation and Forestry, Yezin, Nay Pyi Taw 05282, Myanmar

**Keywords:** Kachin state, *Trivalvaria costata*, Annonaceae, field expedition, Myanmar

## Abstract

*Trivalvaria rubra* and *Trivalvaria casseabriae*, two new species of Annonaceae from Putao, Kachin State, Myanmar, are here described and illustrated. They are morphologically similar to *T. costata* and *T. macrophylla*. The major differences between them are outlined and discussed. A diagnostic key to the species of *Trivalvaria* is provided.

## Introduction

*Trivalvaria* (Miq.) Miq. (Miquel, 1865) is a small genus with six species now recognised, which are mainly distributed in Indochina and Southeast Asia (Scheffer 1869, Das Debika 1968, Kessler 1993, van Heusden 1997, Li and Gilbert 2011, Chatrou et al. 2012). The genus belongs to the subfamily Annonoideae Raf. tribe Miliuseae Hook. f. & Thomson (Chatrou et al. 2012). The monophyly of *Trivalvaria* as well as its sister group relationship with *Marsypopetalum* Scheff., is confirmed with strong support; it was considered very close to *Marsypopetalum* and is characterised by morphological synapomorphies such as extra-axillary inflorescences, short pedicels (less than 1 cm long) and a single, basal ovule in each carpel and hence a solitary seed in each monocarp (Xue et al. 2011, Chaowasku et al. 2014). To date, there are currently two species *Trivalvaria dubia* (Kurz.) J. Sincl. and *T. macrophylla* Miq. recorded in Myanmar (Kress et al. 2003), the former now treated as a synonym of *T. costata* (Hook. f. & Thomson) I. M. Turner (Turner 2009).

Since 2014, repeated China-Myanmar joint field expeditions have been carried out to survey plant diversity in Northern Myanmar, specimens of *Trivalvaria* being found in Putao, Kachin state. Based on a detailed examination of the morphological and anatomical characters of the material and possible closely similar species (van Heusden 1997, Turner 2009, Gardner et al. 2015), it was concluded that the specimens collected in Myanmar belong to species new to science and these are hereby described and illustrated.

## Material and methods

Measurements and morphological character assessments of the two possible new species *Trivalvaria rubra* and *Trivalvaria casseabriae* were examined based on dried specimens and fresh materials in field observations. They were compared with the morphologically similar species *T. costata*, *T. macrophylla* and *T. nervosa*, with affinities inferred using descriptions, type specimens and other herbarium specimens (van Heusden 1997, Turner 2009, Li and Gilbert 2011, Gardner et al. 2015). Protologues and images of type specimens were gathered from JSTOR Global Plants (http://plants.jstor.org). Conservation status evaluations of the new species were based on the International Union for Conservation of Nature guidelines (IUCN 2012).

## Taxonomic treatment

### Trivalvaria rubra

Taxon classificationPlantaeMagnolialesAnnonaceae

Y.H.Tan, S.S.Zhou & B.Yang sp. nov.

urn:lsid:ipni.org:names:60475911-2

Figure 1

#### Diagnosis.

*Trivalvaria rubra* is similar to *Trivalvaria costata* in flower size and petal shape and size and also shares similarities with *T. macrophylla* in leaf shape, but can be distinguished by its pink flowers, androdioecious, petals spreading, outer petal lanceolate to narrowly oblong, 14–20 × 4–6 mm, inner petal 17–25 × 4–7 mm, oblong-ovate to ovate-triangular.

#### Type.

MYANMAR. Kachin State, Putao District, on the way from Nanmti to Nahsihbo, 27°24'29"N, 97°39'59"E, 890 m a.s.l, 16 May 2017, *Myanmar Exped. 1801* (holotype, HITBC!; isotype, RAF!).

#### Description.

**Figure 1.**
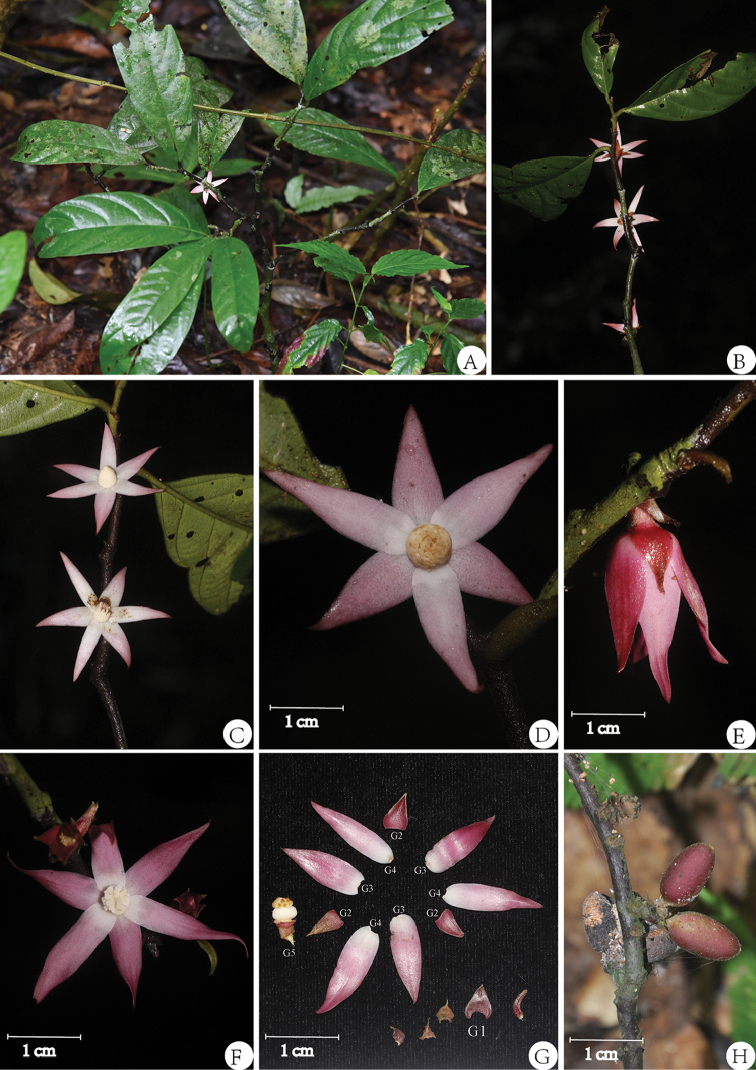
*Trivalvaria rubra* Y.H.Tan, S.S.Zhou & B.Yang, sp. nov. **A** Habitat **B** Branch with flowers (adaxial view) **C** Branch with flowers (abaxial view) **D** Male flower (abaxial view) **E** Flower bud (side view) **F** Female flower (abaxial view) **G** Flower (G1 Bracts; G2 Sepals; G3 Outer petals; G4 Inner petals; G5 Androphore) **H** Fruit. Photographed by Y.H. Tan, H.B. Ding and B. Yang.

Shrubs up to 1.2 m high. Young twigs densely to very sparsely pubescent, older twigs glabrous to pubescent. Leaves subcoriaceous, glabrous above, sparsely pubescent beneath, obovate to narrowly elliptic or oblong-lanceolate, 13.5–27.5 × 4.2–10.5 cm, base cuneate to obtuse, apex attenuate to acuminate or acute, sometimes retuse, midrib sunken above, prominent beneath, sparsely pubescent, lateral veins 9–11 pairs, faintly distinct above, prominent beneath, smaller veins faintly prominent beneath. Petiole 5–10 mm long, 2–3 mm thick, pubescent to glabrous. Flowers pink, androdioecious 2.6–2.8 cm in diam., extra-axillary or ramiflorous, solitary or sometimes in pairs, Bracts 2–4 (Fig. 1.G1), triangular to ovate, 3–6 × 2.5–5 mm, pubescent outside. Pedicel 2–3 mm long, pubescent. Sepals 3 per flower (Fig. 1.G2), free or sometimes shortly connate, triangular to triangular-ovate, 6–9 × 3–6 mm, pubescent to densely pubescent outside, glabrous inside, base rounded, apex acute to acuminate. Petals 6 per flower in two whorls, sub-equal, imbricate, spreading, outer petals (Fig. 1.G3) lanceolate, or oblong-ovate to ovate-triangular, 14–20 × 4–7 mm, pubescent to sparsely pubescent outside, glabrous inside, base rounded to obtuse, apex acute to acuminate; inner petals (Fig. 1.G4) lanceolate or narrowly oblong, 17–25 × 4–6 mm, sparsely pubescent outside, glabrous inside, base rounded to obtuse, apex acute to acuminate. Stamens numerous, ca. 2 mm long, apex shield-like, sometimes tongue-shaped in outer whorl, glabrous; torus triangular conical. Carpels several or many, 15–25 per flower, ovary densely hairy, stigma more or less subglobose, pubescent. Fruiting pedicel 3–4 mm long. Monocarps green, pink to red, ca. 5–10 per fruit, ellipsoid or oblong, 15–18 × 7–10 mm, sparsely pubescent, stipe 2–5 mm, pericarp thin. Seed one per monocarp.

#### Phenology.

Flowering in May to June and fruiting occurs from June to December.

#### Etymology.

The species epithet refers to its pink flower.

#### Distribution and habitat.

*Trivalvaria rubra* is hitherto known from the type locality of Putao, Kachin state in Northern Myanmar, it is a small shrub that grows in the understory in tropical dipterocarpa forests, the dominant tree species are *Shorea assamica*, *Dysoxylum mollissimum*, *Aglaia elaeagnoidea*, *Garcinia* spp., *Goniothalamus peduncularis*, *Antidesma* spp., shrub species are *Hymenandra wallichii*, *Ardisia* sp., *Zingiber* sp., at an elevation of ca. 600–900 m a.s.l.

#### Conservation status.

*Trivalvaria rubra* was collected on the way from Namti to Nahsihbo, Putao, Northern Myanmar, this area being difficult to travel due to its rugged terrain. At least three populations and ca. 60 individuals per population have been discovered. Currently, the locality is not legally protected and, although young individuals were recorded in field, the fragmented habitat and continuous logging severely threaten its survival. At present, it is suggested that it be considered as ‘Vulnerable’ (VU) on the basis of current IUCN Red List Categories and Criteria (IUCN 2012).

#### Specimen examined

(paratypes). MYANMAR. Kachin State, Putao District, near Nahsihbo village, 27°24'36"N, 97°36'26"E, 970 m a.s.l, 11 Dec. 2017, *Myanmar Exped. 3373* (HITBC!); Putao District, near Namti village, 27°24'43"N, 97°39'56"E, 820 m a.s.l, 15 Dec. 2017, *Myanmar Exped. 3698* (HITBC!).

### Trivalvaria casseabriae

Taxon classificationPlantaeMagnolialesAnnonaceae

Y.H.Tan, S.S.Zhou & B.Yang sp. nov.

urn:lsid:ipni.org:names:60475912-2

Figure 2

#### Diagnosis.

*Trivalvaria casseabriae* is similar to *Trivalvaria argentea* in leaf shape, but can be distinguished by its larger flower size and outer petals equal to inner petals, petals 6–10 × 3–5 mm (vs. 2 × 3 mm), 2–2.5 times as long as wide and elliptic to ovate-elliptic.

#### Type.

MYANMAR. Kachin State, Putao District, Wasadam to Upper Shankhaung, 27°26'42"N, 97°14'27"E, 850m a.s.l, 21 May 2017, *Myanmar Exped. 2379* (holotype, HITBC!; isotype, RAF!).

#### Description.

**Figure 2.**
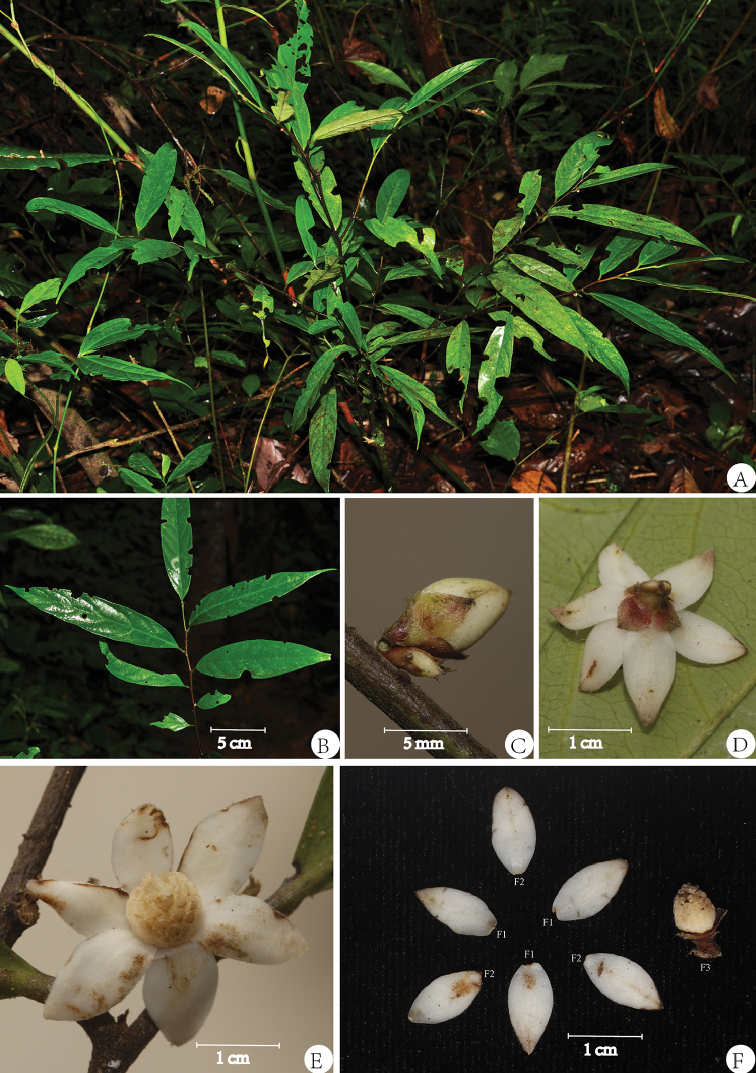
*Trivalvaria casseabriae* Y.H.Tan, S.S.Zhou & B.Yang, sp. nov. **A** Habitat **B** Branch **C** Flower buds (side view) **D** Flower (adaxial view) **E** Flower (abaxial view) **F** Flower (F1 Outer petals; F2 Inner petals; F3 Androphore). Photographed by Y.H. Tan, H.B. Ding and B. Yang.

Shrubs up to 1.5 m high. Young twigs pubescent, older twigs glabrous to sparsely pubescent. Leaves subcoriaceous, glabrous above, sparsely pubescent beneath, lanceolate to oblong, 12.5–24.5 × 2.5–5.5 cm, base cuneate to obtuse, apex acuminate to caudate, midrib immersed above, prominent beneath, sparsely pubescent, lateral veins 5–7 pairs, immersed and faintly distinct above, prominent beneath, smaller veins faintly prominent beneath. Petiole 3–8 mm long, 1–3 mm in diameter, pubescent. Flowers white, androdioecious, 14–20 mm in diam., solitary or in pairs between leaf axils (extra-axillary), rarely ramiflorous. Bracts 1–2, triangular to ovate-triangular, 2–3 × 1–2 mm, pubescent to densely pubescent outside. Pedicel 2–3 mm long, pubescent. Sepals 3 per flower, free or sometimes shortly connate, ovate to broadly ovate, 2–3.5 × 2–3 mm, pubescent outside, puberulous inside and apex acute to obtuse, base rounded. Petals 6 per flower in two whorls, imbricate, spreading, subequal, outer petals (Fig. 2.F1) elliptic to ovate-elliptic, 6–10 × 4–5 mm, sparsely pubescent outside, puberulous inside, base rounded to obtuse, apex acute to obtuse; inner petals (Fig. 2.F2) elliptic to ovate-elliptic, 6–10 × 3–5 mm, sparsely pubescent outside, puberulous inside, base rounded to obtuse, apex acute. Stamens numerous stamens, ca. 2 mm long, apex shield-like, sometimes tongue-shaped in outer whorl, glabrous; torus triangular conical. Carpels 6–10 per flower, with globose stigma, pubescent. Monocarps and seeds not seen.

#### Phenology.

Flowering at May to July.

#### Etymology.

The specific epithet is derived from the abbreviation of Southeast Asia Biodiversity Research Institute, Chinese Academy of Sciences (CAS-SEABRI); noun in apposition. The name is dedicated to its three-part mission, viz., (a) Serve China’s “the Silk Road Economic Belt and the 21st Century Maritime Silk Road” initiative; (b) Integrate efforts by CAS and international institutes to organise professional research groups and train researchers in Southeast Asian countries; and (c) Provide support to all domestic and international partners.

#### Distribution and habitat.

*Trivalvaria casseabriae* is only known from the type locality of Putao, Kachin state in Northern Myanmar, where it grows in the understory of tropical montane broadleaf forests, the dominant tree species being *Altingia excels*, *Dysoxylum* spp., *Garcinia* spp., *Elaeocarpus* spp., at an elevation of ca. 700–900 m.

#### Conservation status.

Although *Trivalvaria casseabriae* was collected on the way from Wasadam to Upper Shanhkaung, Putao District, Northern Myanmar, only one population and less than 50 individuals, has been discovered. Potential populations and more individuals may be found in future field surveys. Currently, the locality is not legally protected and, although young individuals were recorded in field, the fragmented habitat and continuous logging severely threaten its survival. At present, it is suggested that it be considered as ‘Vulnerable’ (VU) on the basis of current IUCN Red List Categories and Criteria (IUCN, 2012).

#### Specimen examined

**(paratypes).** MYANMAR. Kachin State, Putao District, on the way from Wasadam to Upper Shankhaung, 27°26'39"N, 97°14'23"E, 800 m a.s.l, 21 May 2017, *Myanmar Exped. 2389* (HITBC!), Putao District, Upper Shanhkaung, 27°26'30"N, 97°14'26"E, 680 m a.s.l, 28 April 2016, *Myanmar Exped. 201614* (HITBC!).

##### Key to the species of the genus *Trivalvaria*

**Table:** 

1	Flowers minute, less than 10 mm in diam.; inner petals ca. 2–4 mm long	**2**
–	Flowers conspicuous, more than 10 mm in diam.; inner petals more than 5 mm long	**3**
2	Leaf blade obovate to oblong; petals glabrous inside; monocarps ellipsoid-oblong, 14–20 × 7–10 mm	***T. argentea***
–	Leaf blade lanceolate; petals puberulous inside; monocarps subglobose, 9–10 mm in diam.	***T. kanjilalii***
3	Petals pubescent or puberulous inside	**4**
–	Petals glabrous inside	**5**
4	Tree to 15 m, elliptic-oblong to oblong-lanceolate, 2.8–3.5 times as long as wide	***T. nervosa***
–	Shrub up to 1.5 m, leaf blade lanceolate to oblong, 4.5–5 times as long as wide	***T. casseabriae***
5	Petals densely hairy outside, inner petals of mature flowers connivent, less than 1.5 times as long as wide	***T. macrophylla***
–	Petals pubescent or hairy outside, inner petals of mature flowers spreading, 2–4 times as long as wide	**5**
6	Flowers pink	***T. rubra***
–	Flowers white	**6**
7	Monocarps subglobose; inner petals oblanceolate, tip triangular to ligulate, ca. 2 times as long as wide; leaf blade less than 2.5 times as long as wide	***T. carnosa***
–	Monocarps elliptic-oblong; inner petals oblanceolate, elliptic to narrowly oblong, 3–4 times as long as wide; leaf blade 3 times as long as wide	***T. costata***

## Discussion

Also as a genus, *Trivalvaria* was revised and mentioned by several experts (Heusden 1997, Gardner et al. 2015), but it is still poorly understood due to the very limited collections and poorly known taxonomic information. For this study, the authors have tried to combine the original description and field observations to present a brief approved taxonomic characters (Table 1).

**Table 1.** Morphological comparison of key characters and distribution in *Trivalvaria rubra*, *T. casseabriae* and the similary taxa. Morphological characters of *T. costata*, *T. macrophylla*, *T. nervosa*, *T. argentea* following Heusden (1997) and Gardner et al. (2015), *T. kanjilalii* following Das (1968), *T. carnosa* following Scheffer (1869) , Teijsmann (1863) & our field observation in Xishuangbanna Tropical Botanical Garden (XTBG).

Character	*Trivalvaria rubra*	*T. casseabriae*	*T. costata*	*T. macrophylla*	*T. nervosa*	*T. argentea*	*T. kanjilalii*	*T. carnosa*
Habitat	shrub up to 1.2 m	shrub up to 1.5 m	shrub to 3 m	tree or shrub up to 12 m	tree to 15 m	shrub	shrub 2–3 m	shrub to1m
Leaf blade	obovate to narrowly elliptic or oblong-lanceolate, 13.5–27.5 × 4.2–10.5 cm	lanceolate to oblong, 12.5–24.5 × 2.5–5.5 cm	narrowly elliptic to obovate or oblong-lanceolate, 12–20 × 4–7 cm	elliptic-oblong to oblanceolate 9(–16)–(22–)30 × 3–10 cm	elliptic-oblong to oblong-lanceolate, 17–37 × 5–13 cm	obovate to oblong, 9–20 × 2.5–7 cm	lanceolate 12–15 × 3–4 cm	ovate-oblong, 15–17 × 6.5–7 cm
Flowers	pink, polygamous (male and bisexual), 26–28 mm in diam.	white, male and bisexual, 14–20 mm in diam.	white, male and bisexual, ca.12–24 mm in diam.	white to pale brownish creamy, bisexual, ca. 12 mm in diam.	white, polygamous (male and bisexual), ca. 14–26 mm in diam.	minute, ca. 6–8 mm in diam.	bisexual, ca. 5–6 mm in diam.	white, bisexual, 14–20 mm in diam.
Sepals	triangular to triangular-ovate, 6–9 × 3–6 mm	ovate to broadly ovate, 2–3.5 × 2–3 mm	triangular to broadly ovate, 2–3.5 × 1.5–4 mm	broadly ovate or triangular, 3–4 × (4–)5–6 mm	broadly ovate, 1.5–3 × 2–3.5 mm	broadly triangular-ovate, 1.5 × 2 mm	broadly ovate, 3–4 × 4 mm	broadly ovate, 2–3 × 2 mm
Petals	glabrous inside	puberulous inside	glabrous or downy inside	glabrous inside	pubescent inside	glabrous inside	puberulous inside	glabrous inside
Outer petals	lanceolate, or oblong-ovate to ovate-triangular, 14–20 × 4–7 mm	elliptic to ovate-elliptic, 6–10 × 4–5 mm	oblong-lanceolate, elliptic-oblong, 4–8(–12) × (1–)2–4 mm	broadly ovate or or triangular, 4–8 × 4–7 mm	obovate to elliptic- oblong, (6–)8–15 × 3.5–10 mm	ca. 2 mm long	ovate, 2.5–3 × 2.5 mm	broadly ovate, 5–6 × 4–5 mm
Inner petals	spreading, lanceolate or narrowly oblong, 17–25 × 4–6 mm	spreading, elliptic to ovate-elliptic, 6–10 × 3–5 mm	spreading, oblanceolate, elliptic to narrowly oblong, 4–12 × 1–4 mm	connivent, broadly ovate to broadly elliptic, or broadly triangular-ovate, 5–13 × 4–10 mm	spreading, obovate to elliptic-oblong, (5–)7–17 × 3–9 mm	ca. 2 mm long	tip incurved, more or less rhomboid, 3–4 × 2.5–3 mm	oblanceolate, tip triangular, 10–12 × 5–6 mm
Monocarps stipe	2–5 mm	unknown	1–6 mm	2–6 mm	9–(25–)30 mm	3–6 mm	5–6 mm	5–6 mm
Monocarps	ellipsoid or oblong, 15–18 × 7–10 mm	unknown	elliptic-oblong, 12–24 × 6–10 mm	ovoid to ellipsoid or oblong, 14–20 × 7–10 mm	oblong, 20–25 × 13–15 mm	ellipsoid-oblong, 14–20 × 7–10 mm	subglobose, 9–10 mm in diam.	subglobose, 10–12 × 8–10 mm
Distribution	Myanmar	Myanmar	Southeast Asia, China (Hainan)	S. Thailand, Malaya, Sumatra, Java, Borneo	S. Thailand and Malaysia	NE India, Bangladesh	E India	Java

## Supplementary Material

XML Treatment for Trivalvaria rubra

XML Treatment for Trivalvaria casseabriae
